# Mummy Material Can Promote Differentiation of Adipose Derived Stem Cells into Osteoblast through Enhancement of Bone Specific Transcription Factors Expression

**DOI:** 10.15171/apb.2018.053

**Published:** 2018-08-29

**Authors:** Maryam Eyvazi, Raheleh Farahzadi, Nahid Karimian Fathi, Mohammad Karimipour, Jafar Soleimani Rad, Azadeh Montaseri

**Affiliations:** ^1^Stem Cell Research Center, Tabriz University of Medical Sciences, Tabriz, Iran.; ^2^Hematology and Oncology Research Center, Tabriz University of Medical Sciences, Tabriz, Iran.; ^3^Biochemistry Department, Faculty of Medicine, Tabriz University of Medical Sciences, Tabriz, Iran.; ^4^Anatomical Sciences Department, Faculty of Medicine, Tabriz University of Medical Sciences, Tabriz, Iran.

**Keywords:** Osteoblast, Adipose derived stem cells, Differentiation, Transcription factor

## Abstract

***Purpose:*** Application of Mummy material for treatment of different diseases such as bone fracture, cutaneous wounds and joint inflammation has been advised since hundred years ago in Persian traditional medicine. Due to the claims of indigenous people and advice of traditional medicine for application of this material in healing of bone fractures, this study has been designed to evaluate whether Mummy material can promote the differentiation of mesenchymal stem cells into osteoblasts and enhance the expression of bone specific genes and proteins.

***Methods:*** Adipose derived stem cells (ASCs) at fourth cell passage were divided into control, osteogenesis group (received osteogenic medium), Mummy group (received Mummy at concentration of 500 µg/ml). ASCs in the fourth group were treated with both osteogenic medium and Mummy (500µg/ml). Cells in all groups were harvested on days 7, 14 and 21 days for further evaluation through Real time RT-PCR, Von kossa staining, Immunocytochemistry and flowcytometery.

***Results:*** Treatment of ASCs with Mummy at concentration of 500µg/ml promotes the expression level of Osteocalcin, RUNX-2 and β1-integrin genes in different time points but that of the Osterix did not changed. Furthermore the expression of Osteocalcin protein enhanced significantly in ASCs treated with Mummy detected by Immunocytochemistry and flowcytometery technique compared to the control groups. The results of this study also showed that treatment of ASCs with Mummy resulted in formation of mineral deposits which was evaluated by Von Kossa staining method.

***Conclusion:*** Obtained data from this study reveals that Mummy is a potent enhancer for differentiation of ASCs into osteoblasts in in vitro system, probably through increasing the level of bone specific genes and proteins.

## Introduction

Bone is a specialized type of connective tissue which is composed of two different cells named as osteoblasts and osteoclasts.^1^ Coordinated balance between activities of these cells is responsible for maintenance of bone homeostasis and remodeling.^2^ Synthesis of organic components of bone matrix is attributed to osteoblasts, while bone resorption occurs through activity of osteoclasts.^3^ Disruption of this precisely regulated balance can result in bone related diseases as osteoporosis which is defined as bone mass reduction.^4^ The prevalence of bone disorders is increasing, in part because of growing of aging population. On the other hand bone defects can also occur due to trauma, surgery, tumor metastasis and metabolic diseases, so finding efficient therapeutic methods for bone defects is of great importance.

Recently, *in vitro* production of bone constructs produced by tissue engineering technique has been introduced as a new strategy for treatment of bone disorders.^5^ Successful tissue engineering can be achieved through application of biodegradable scaffolds seeded by adequate cells such as mesenchymal stem cells or osteoblasts. Mesenchymal stem cells are multipotent and undifferentiated cells which exhibits potential for differentiation into variety of cells including chondrocytes, adipocytes and osteoblasts.^6^ These cells can be harvested from different sources such as bone marrow, umbilical cord Wharton's jelly, muscular and also adipose tissue.^7^ Among different sources, adipose tissue has been introduced as a suitable candidate because of its wide distribution through the body, easy access, less morbidity and great number of stem cells which can be extracted from it.^8^ During embryonic development, osteoblasts are derived from mesenchymal stem cells. Osteogenic differentiation of these cells is regulated by different specific transcription factors such as osterix and RUNX-2(Runt-related transcription factor-2).^4^ These key factors modulate the mesenchymal stem cells commitment into osteoblasts through expression of bone marker genes such as Alkaline phosphatase (Alp), collagen type, osteocalcin and osteopontin.^9-12^

As it has been previously noted, in recent decades bone tissue engineering has been emerged as a successful technique for treatment of bone defects.^13^ For improving these cell-based therapeutic methods, growth factors and cytokines have been applied to enhance bone repair.^14,15^ Despite of advantages, considering to the high costs and rapid degradation the clinical application of these factors is limited.^16^ Therefore there is a continuing and urgent need to develop alternative agents with higher efficacy, less side effects and lower costs. In recent decades, researcher's focus has switched on application of natural compounds derived from several plants with positive effects on bone repair.^17^ Over hundreds of years, in Persian traditional medicine, Mummy has been used as a healer for different diseases as bone fractures, joint inflammation, cutaneous wounds and gastric ulcers.^18^ Mummy which is named also Mumnaye by local people is a dark brown, semi-solid and pitch like material found in some cracks of rare caves and secreted due to oil oxidation.^19^ Chemical analysis revealed the presence of calcium, phosphate, carbonate, magnesium, oxygen, nitrogen and also polysaccharide in this material. A clinical investigation performed by Dehghan et al.(2012) showed that Mummy can enhance the healing process of tibial and femoral fractures and reduce the complications.^18^

In consideration to the probable effects of Mummy in acceleration of bone healing claimed by indigeneous people, the present study was designed to evaluate whether Mummy can enhance the differentiation of Adipose derived mesenchymal stem cells (ASCs) into osteoblast through expression of major transcription factors as RUNX-2 and osterix and also osteogenesis-related marker named osteocalcin.

## Materials and Methods

### Adipose Tissue collection and isolation of mesenchymal stem cells

Adipose tissue samples were obtained from patients undergoing laparotomy. Isolation of mesenchymal stem cells from adipose tissue was performed as described previously by Fathi et al. (2017).^20^ After transferring to the culture lab, samples were washed ascetically using phosphate buffered saline (PBS) containing 1% Penicillin/streptomycin (P/S) for 3 times. Mechanical digestion was performed by mincing the adipose tissue samples using sterile scalpel, and then followed by enzymatical digestion. Through incubation of samples in collagenase type I (0.2% in free DMEM) per each gram while shaking in water bath for about 60-90 minutes. After digestion of main pieces, DMEM medium containing 10% fetal bovine serum(FBS) was added for neutralizing the enzyme activity, and then filtered through 70µm cell strainer for isolation the undigested particles. Obtained cell suspension was centrifuged for 10 minutes at 1500 rpm and then cells were plated at density of 5×10^5^/ T75 flasks in 5% co_2_ and 37°c.After reached about 70% confluency, cells were trypsinized and expanded. Adipose derived stem cells (ASCs) at fourth passage were used for this study.

### Preparation of Mummy

Mummy was purchased from local botanical markets in Kermanshah, Iran. Mummy was used at concentration of 500µg/ml in this study; it should be noted that the selection of Mummy material concentration was done based on the previous evaluations has been performed by our research team.^21^ Mummy can be solved completely in water, so it was solved in DMEM culture medium and then passed through syringe filter (0.22µm) for sterilization.

### Study design

To evaluate whether Mummy can affect osteogenesis in ASCs, cells at 4^th^ passage were divided into different groups as: control (group I), which received serum DMEM(0/05% FBS), osteogenesis group (group II) which treated with osteogenic induction medium containing DMEM base medium, 10^-7^ mol dexamethasone (Sigma,cat.No.D8893, Germany), 10mmol β-glycerophosphate (sigma,cat.No.9422, Germany) and 50µmol ascorbake-2-phosphate (sigma,cat.No.A5960, Germany), ASCs in third group (group III) were treated with osteogenic medium and Mummy at concentration of 500µg/ml and in fourth group (group IV), ASCs received Mummy (500µg/ml). Cells were harvested on 7, 14 and 21 days for further evaluation through Real time RT-PCR, Von kossa staining, Immunocytochemistry and flowcytometery.

### Gene expression using Real time reverse transcriptase polymerase chain reaction (RT-PCR) technique

The gene expression profile for Osterix, RUNX-2, Osteocalcin and β-1 integrin in different groups was detected by Real time RT-PCR technique.

For performing this method, total cellular RNA was extracted using YTA mini kit (cat.No:YT9065,Taiwan) according to instructions. Briefly cell lysis was done using RB buffer and then mixture was transferred to the collection tube. After centrifugation, 70% ethanol was added to each sample. Subsequently RNase-free H_2_O was added to samples to obtain RNA. Approximately 1000µg/ml of total obtained RNA was used for cDNA synthesis using reverse transcription kit (Takara, RR0371, Japan).In the next step using PCR Rotorgene 6000. (Corbett, 010755, Australia) and SYBR Green PCR master mix (Takara,RR20 L, Japan),Real time RT-PCR reactions were performed. Table 1 shows different time and annealing temperatures for each gene.

**Table 1:** Primer sequences used in Real Time RT-PCR

**Gene**	**Primer**	**Annealing temperature**
Osterix-F	TAGGACTGTAGGACCGGAGC	60.11°C for 30 sec
Osterix-R	TGTCCCGAGTCTCTCCTCTC	59.75 °C for 30 sec
Integrin subunit beta 1-F	GCCGCGCGGAAAAGATGAAT	62.02‏°C for 30 sec
Integrin subunit beta 1-R	CACAATTTGGCCCTGCTTGTA	61.75‏°C for 30 sec
Osteocalcin -F	TCCTTTGGGGTTTGGCCTAC	59.89 °C for 30 sec
Osteocalcin -R	CCAGCCTCCAGCACTGTTTA	59.96 °C for 30 sec
RUNX2-F	CCACCGAGACCAACAGAGTC	60.04 °C for 30 sec
RUNX2- R	TCACTGTGCTGAAGAGGCTG	59.97 °C for 30 sec

### Von kossa staining method for evaluation of mineralized matrix deposition

Monolayer cultivated ASCs were stained with Von kossa for observing the mineralized depositions. For performing the staining protocol, cells in different groups were fixed by ethanol and then washed 3 times with distilled water (D/W). Then incubation with silver nitrate was performed and followed by incubation in pyrogalic acid 1% for 5 minutes. After washing with D/W, cells were fixed with sodium thiosulfate (5%) and finally counterstained with nuclear fast red solution (0/1%) for observing the nuclei.

### Immunocytochemistry staining technique

A total of 200 × 10^3^ cells were seeded in an 8-wells cell culture chamber slide. After one day of culture, the cells were washed with PBS and then fixed in 4% paraformaldehyde for 30 minutes at room temperature. After fixation, the cells were washed twice with PBS, and once with PBS and 3% FBS. The cells were incubated overnight at 4ºC with a 1:100 dilution of mAbs against osteocalcin (Human/Rat Osteocalcin Antibody MAB1419, R&D Systems, UK) in PBS. The cells were then washed with PBS, and incubated with a 1:500 dilution of the biotin-conjugated mouse monoclonal IgG1 antibody against rats in PBS and 1% BSA overnight. After washing with PBS, a 1:500 dilution of secondary antibody (goat anti-mouse IgG-PE, sc-3738, USA) was added for 2 hours at 37°C. The cells were washed three times with PBS and the nuclei were stained with DAPI for 30 seconds. After three washes with PBS, the fluorescent cells were visualized under the fluorescence microscop.^22^

### Flow-cytometric analysis 

Approximately 10^6^ cells were washed twice with PBS containing 3% FBS (washing buffer), were blocked by blocking reagents, were fixed with FCM Fixation Buffer and were incubated with osteocalcin primary antibody (Human/Rat Osteocalcin Antibody MAB1419, R&D Systems, UK) for 30 minutes on ice. After terminating the incubation time, the cells were then washed with washing buffer, and incubated with a PE-conjugated gout monoclonal IgG1 antibody against mouse for 30 min on ice. hADSCs were washed with washing buffer again and FACSCalibur system (BD Biosciences, San Diego, CA) was used for determing the osteocalcin protein expression as intracellular staining. Data were analyzed using with the FlowJo software (version 6.2).

### Statistical analysis

Statistical difference between different groups was determined by Two-Way ANOVA method and followed by t-test post test. The results are shown as the mean ± SD of a representative experiment performed three times. Data shown are representative of three independent experiments.

## Results

### Effects of Mummy on gene expression in ASCs differentiated into osteoblasts

**Figure 1:**
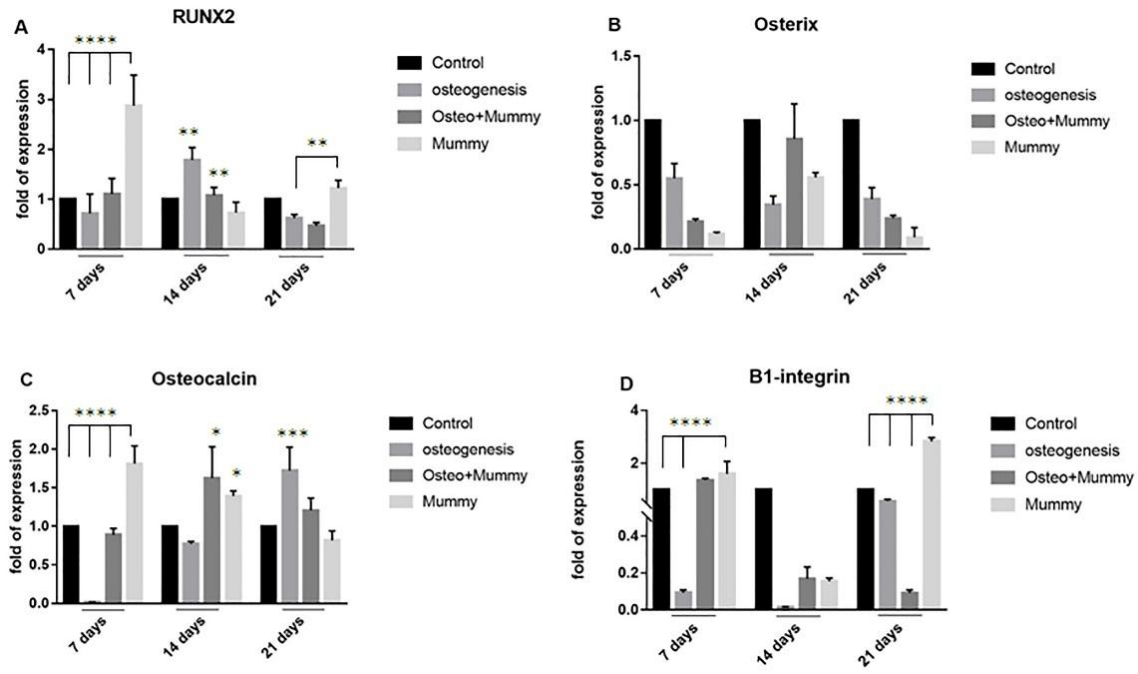
The effect of Mummy material on ASCs differentiation into osteoblasts evaluated by Real time RT-PCR. A) Expression of RUNX-2, B) Osterix, C) Osteocalcin and D) β1- Integrin. Following treatment of ASCs with Mummy, the expression level of RUNX-2 increased significantly on days 7 and 21 compared to the control and also osteogenesis groups. The expression of osteocalcin also increased significantly after 7 and 14 days of treatment with Mummy material compared to the control group. Furthermore, a significant increase in the level of β1-integrin gene expression was found after treatment of ASCs with Mummy material on 7 and 21 days of culture period, but the expression level of Osterix did not affect significantly between different groups. *P<0.01, **P<0.001, ***P<0.0001 and ***P<0.00001

The effect of Mummy on differentiation of ASCs to osteoblasts were evaluated through expression of osteocalcin, RUNX-2 and osterix genes, also β_1-_integrin gene expression was evaluated to examine whether Mummy can enhance the cell-cell and cell-matrix adhesion which is crucial for cell survival. Figure 1-A reveals the expression level of transcription factor RUNX-2 which has crucial role in regulation of bone development. As it can be found from this Figure, treatment of ASCs with Mummy at concentration of 500µg/ml (group IV) resulted in a significant increase in RUNX-2 expression compared to control and osteogenic induced cells after 7 and 21 days of culture, but not at 14 days. After 14 days, the expression level of this transcription factor increased significantly in ASCs induced with osteogenic medium (group II) and also cells co-treated with osteogenic medium and Mummy( 500µg/ml) (group III) compared to the control cells (P<0.001).

In this study the expression level of osterix as an essential marker for bone formation also was evaluated to find whether treatment of ASCs with Mummy can enhance its expression. Figure 1-B shows that there isn’t any increase in osterix expression in ASCs treated with Mummy or osteogenic medium compared to the control group at different time points. Another gene which its expression was evaluated in this investigation was osteocalcin. Figure 1-C reveals that in the presence of Mummy (500µg/ml) the expression level of osteocalcin increased significantly compared to the control groups on 7 and 14 days of culture but on 21 days there isn’t any increase.

The expression level of β1-integrin in different groups at different time points has shown in Figure 1-D. As it can be understood, treatment of ASCs with Mummy resulted in a significant increase in β1-integrin expression compared to the other groups after 7 and 21 days of culture period but not at 14 days.

### Light microscopy

**Figure 2:**
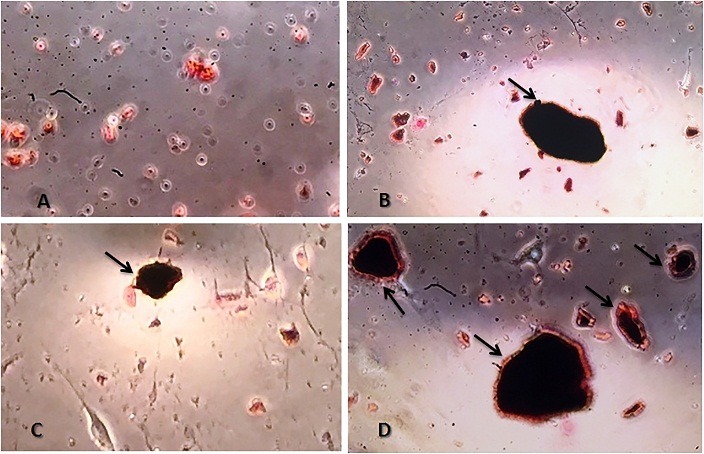
Von Kossa staining method for investigating the calcium deposition in different groups. A) Control cells, B) Osteogenic differentiation medium, C) osteogenic differentiation medium and Mummy, and D) Mummy-treated cells. In untreated (control) group, no calcium deposition was observed but strong deposition of calcium can be seen in cells treated with osteogenic medium (B) and also cells which received Mummy (D). 400x

Von kossa staining method was performed to evaluate mineral deposition in different groups after 7 days of culture. As it can be found in Figure 2A, in control cells, no calcium deposition was observed. In contrast to control ASCs, cells which received osteogenesis medium (group II) or induced with osteogenic medium and treated synchronizely with Mummy at concentration of 500µg/ml (group III) were stained positive for calcium deposition (Figure 2B and C). Figure 2-D shows mineral deposition in ASCs treated with Mummy (group IV), as it can be understood strong calcium depositions were detected in ASCs treated with Mummy at concentration of 500 µg/ml.

### Detection of osteocalcin protein using Immunocytochemistry and flowcytometery method

**Figure 3:**
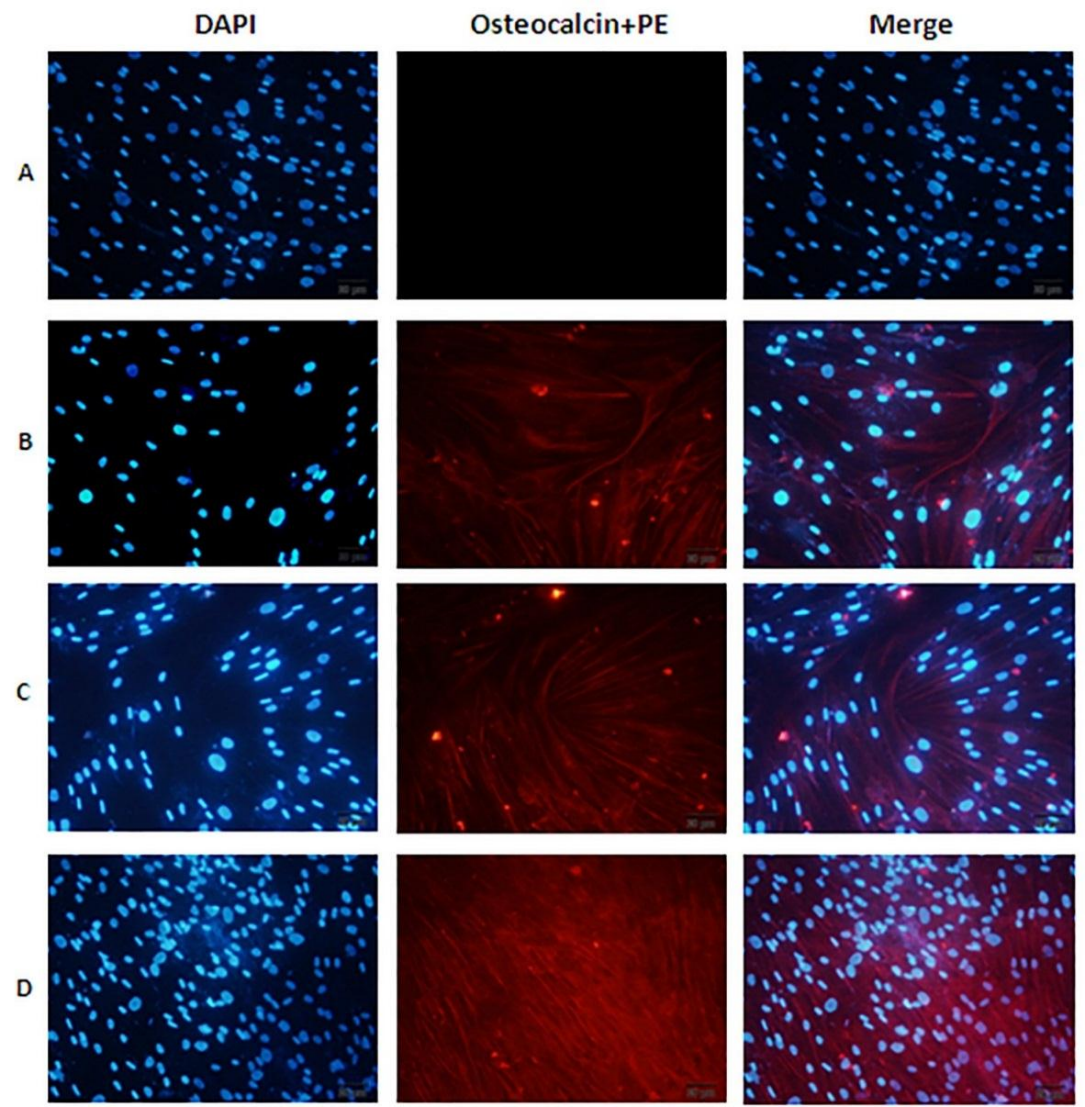
Immunofluorescence staining for the expression of osteocalcin as intracellular marker of adipose tissue derived mesenchymal stem cells (ADSCs) in the presence of different culture medium; (A) DMEM culture medium, (B) Osteogenic differentiation medium, (C) Osteogenic differentiation medium+Mummy, (D) Mummy dissolved in DMEM (500µg/ml). At the end of 7 days treatment with each culture medium, ADSCs were labeled using PE-conjugated anti-osteocalcin (red) antibodies; nuclei were stained blue by 4′,6-diamidino-2-phenylindole (DAPI)

**Figure 4:**
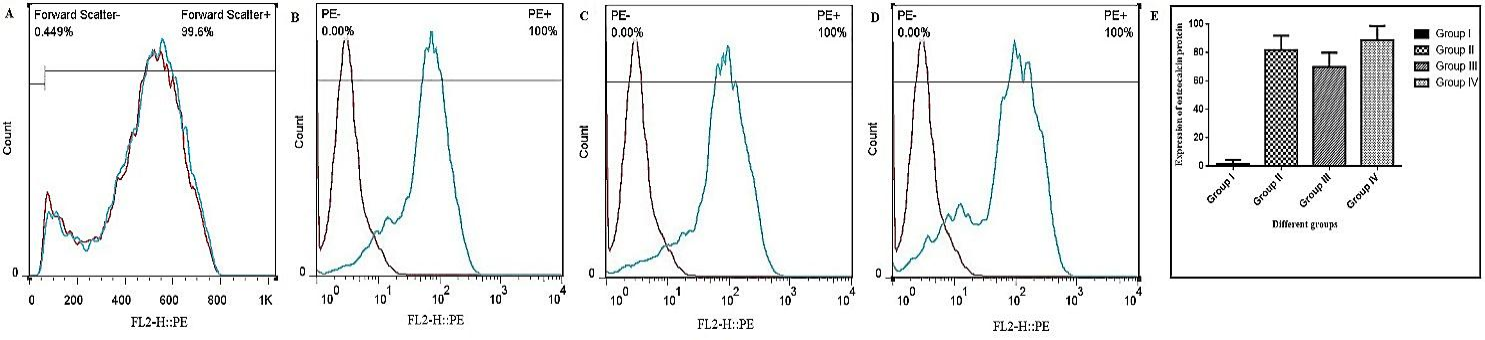
The expression of the osteocalcin intracellular marker of ADSCs as analyzed by flow cytometry. ADSCs were treated with different culture mudium for 7 days before flow cytometry assay; (A) Osteogenic differentiation medium, (B) Osteogenic differentiation medium+Mummy, (C) Mummy; Each antibody was tested individually and the isotopes controls were used as the negative control (red lines) in this experiment. (D) The diagram for comparison between groups. One-way ANOVA followed by Dunnett's post hoc test was used to determine the significant difference among groups; the p value was not significant between groups

As shown in Figure 3, immunofluorescence staining on ADSCs in the presence of different culture medium was demonstrated that the majority of the cells were positive for osteocalcin markers in group II (treated with osteogenic differentiation medium), III (received both osteogenic differentiation medium and Mummy) and IV (treated with Mummy 500µg/ml) as compared with group I (control cells). Also, the effect of mummy on osteogenic differentiation of ADSCs was performed by the flow cytometry for osteocalcin protein expression as well as immunocytochemistry method. Flow cytometry graphs (Figure 4) revealed that 83.7%, 69.7% and 88.9% of the ADSCs expressed osteocalcin in group II (Osteogenic differentiation medium), III (Osteogenic differentiation medium+Mummy) and IV (Mummy), respectively. However, the p-value was not significant between groups.

## Discussion

In the present investigation we examined whether Mummy can enhance the differentiation of ASCs into osteoblasts. The result of this study showed that: Treatment of ASCs by Mummy at dose of 500µg/ml increased the expression level of RUNX-2, osteocalcin and β1-integrin in different time points, in the presence of Mummy the expression of osteocalcin protein detected by Immunocytochemistry technique was increased, and treatment of ASCs with Mummy (500µg/ml) resulted in formation of mineral deposition which was detected using Von Kossa staining protocol.

Recently cell-based bone tissue engineering technique has been emerged as a new therapeutic method for bone reconstruction.^23^ For performing a successful tissue engineering on this field focused on finding an ideal cell source.^24^ Identifying mesenchymal stem cells (MSCs) opened a new frontier for skeletal tissue engineering approaches. Adipose derived mesenchymal stem cells(ASCs) with potential for differentiating into chondrocytes, adipocyte and osteoblasts are introduced as a suitable candidate for bone regenerative techniques.^23^ Another factor for enhancing bone regeneration and promoting the differentiation of MSCs into osteoblasts is the use of anabolic growth factors.^14^ Despite of many advantages, application of this factor for tissue engineering approaches has its own limitations such as production costs and short half life, so finding other enhancers with more effectiveness and less cost is of great importance.

In most Asian countries application of natural-based products hold great promise for treatment of different diseases such as bone deformities and fractures.^25^ Since hundreds of years Mummy has been advised for treatment of bone fracture in Persian traditional medicine. Due to unclear underlying mechanisms of this material in fracture healing, we designed this study to investigate whether Mummy can enhance the differentiation of Mesenchymal stem cells into osteoblasts through upregulation of specific bone transcription factors and ECM components.

In this study we understood that treatment of ASCs with Mummy at concentration of 500µg/ml resulted in a significant increase in expression level of RUNX-2, osteocalcin and β1-integrin but that of the osterix didn’t changed.

Runt-related transcription factor 2(RUNX-2) is a key transcription factor that regulates osteoblast differentiation from mesenchymal stem cells and also controls the expression of bone extracellular matrix proteins during osteogenesis.^26^ This transcription factor plays a crucial role in both intramembraneos and endochondral ossification and loss of its function results in severe skeletal deformities.^27^ This knowledge about importance of RUNX-2 led us to examine whether Mummy material can alter the expression of this transcription factor in ASCs. In fact we found that Mummy at concentration of 500µg/ml can promote the expression of RUNX-2 gene significantly on 7 and 21 days. These finding suggest probable involvement of RUNX-2 signaling in differentiation of osteoblasts from ASCs in the presence of Mummy material.

Another transcription factor involved in bone formation is osterix.^28^ This transcription factor has an essential role in differentiation of osteoblast precursors into mature cells and furthermore is expressed during bone repair fracture sites. In this study we investigated whether treatment of ASCs with Mummy can promote the expression level of osterix mRNA.

Obtained data showed that on the evaluated time points the level of osterix gene decreased compared to the control cells.

Additionally in this investigation we found that in the presence of Mummy the expression level of osteocalcin and β1-integrin mRNA increased significantly compared to the control and osteogenic-induced groups. Furthermore using ICC method we found that the expression of osteocalcin protein in Mummy treated ASCs elevated compared to other groups.

Osteocalcin is the most abundant glycoprotein in bone extracellular matrix which can bind to calcium ions.^29,30^ This protein is produced by osteoblasts, odontoblasts and hypertrophic chondrocytes. Having role in regulating the mineralization of bone matrix, osteocalcin is used as a marker for evaluation of osteoblast activity.

Moreover we understood that Mummy can increase significantly the expression level of β1-integrin gene compared to other groups. This transmembrane protein mediates cell-cell and cell-matrix interactions and also involves in maintenance of cell phenotype. Furthermore it has been reported that integrin molecules are involved in regulation of collagen type I synthesis.

Taking toghether, the results of this investigation showed that Mummy material can upregulate the expression of RUNX-2, osteocalcin and β1-integrin mRNA levels in ASCs. More extensive investigation is necessary for better understanding the underlying mechanisms of this material in differentiation of ASCs into osteoblasts.

## Conclusion

As a summary, the results of this study showed for the first time that treatment of ASCs with Mummy material can enhance the expression level of bone-specific markers and promote the deposition of mineral components by ASCs. Further investigation for better understanding the precise mechanisms of this material on intracellular signaling pathways in adipose derived stem cells is necessary.

## Acknowledgments

This article is resulted from the research proposal leading to thesis of Maryam Eyvazi, M.Sc student of Anatomical Sciences and approved by Stem Cell Research center, Tabriz University of Medical sciences, Tabriz, Iran. The authors gratefully acknowledge the research deputy of Tabriz University of Medical Sciences for financial support.

## Ethical Issues

Written consent was taken from all patients and the study protocol was approved by medical ethics committee of Tabriz University of Medical sciences (ethical number: IR.TBZMED.REC.1395.229).

## Conflict of Interest

The authors declare no conflict of interests.
